# Promoter Methylation of the *MGRN1* Gene Predicts Prognosis and Response to Chemotherapy of High-Grade Serous Ovarian Cancer Patients

**DOI:** 10.3389/fonc.2021.659254

**Published:** 2021-06-29

**Authors:** Xiao-fei Li, Hai-yan Sun, Tian Hua, Hai-bo Zhang, Yun-jie Tian, Yan Li, Shan Kang

**Affiliations:** ^1^ Department of Obstetrics and Gynaecology, Hebei Medical University, Fourth Hospital, Shijiazhuang, China; ^2^ Department of Obstetrics and Gynaecology, Affiliated Xing Tai People Hospital of Hebei Medial University, Xingtai, China; ^3^ Department of Molecular Biology, Hebei Medical University, Fourth Hospital, Shijiazhuang, China

**Keywords:** *MGRN1*, HGSOC, methylation, platinum resistance, prognosis

## Abstract

Aberrant DNA methylation is considered to play a critical role in the chemoresistance of epithelial ovarian cancer (EOC). In this study, we explored the relationship between hypermethylation of the Mahogunin Ring Finger 1 (*MGRN1*) gene promoter and primary chemoresistance and clinical outcomes in high-grade serous ovarian cancer (HGSOC) patients. The MALDI-TOF mass spectrometry assays revealed a strong association between hypermethylation of the *MGRN1* upstream region and platinum resistance in HGSOC patients. Spearman’s correlation analysis revealed a significantly negative connection between the methylation level of *MGRN1* and its expression in HGSOC. In vitro analysis demonstrated that knockdown of *MGRN1* reduced the sensitivity of cells to cisplatin and that expression of *EGR1* was significantly decreased in SKOV3 cells with low levels of *MGRN1* expression. Similarly, *EGR1* mRNA expression was lower in platinum-resistant HGSOC patients and was positively correlated with *MGRN1* mRNA expression. Kaplan-Meier analyses showed that high methylation of the *MGRN1* promoter region and low expression of *MGRN1* were associated with worse survival of HGSOC patients. In multivariable models, low *MGRN1* expression was an independent factor predicting poor outcome. Furthermore, low expression of *EGR1* was also been confirmed to be significantly related to the poor prognosis of HGSOC patients by Kaplan-Meier. The hypermethylation of the *MGRN1* promoter region and low expression of *MGRN1* were associated with platinum resistance and poor outcomes in HGSOC patients, probably by altering *EGR1* expression.

## Introduction

Due to the lack of initial symptoms and sensitive screening methods, approximately 70% of women with epithelial ovarian cancer (EOC) are diagnosed at an advanced stage of disease (1, 2); EOC is the most lethal gynecologic malignancy in China (3, 4). Currently, the treatment strategy for patients with advanced EOC is platinum-based chemotherapy following primary debulking surgery (5–7). Although the majority of EOC patients respond well to first-line chemotherapy, most of these patients relapse and develop platinum resistance within 2 years (7, 8). In addition, nearly 20% of patients do not respond at the beginning of chemotherapy (9–11). Therefore, chemotherapy resistance has become an important cause of high mortality among EOC patients.

Resistance to chemotherapeutics, including intrinsic and acquired resistance, is based on highly complex and individually variable biological mechanisms (12). Abnormal methylation of DNA has been considered to play an important role during the development of acquired chemoresistance in EOC patients (13). However, there are currently very few studies about the effect of DNA methylation on the development of intrinsic resistance in EOC patients. In our previous study, reduced representation bisulfite sequencing (RRBS) analysis showed that the promoter region of the Mahogunin Ring Finger 1 (*MGRN1*) gene had abnormal hypermethylation in high-grade serous ovarian cancer (HGSOC) patients with platinum resistance (14). *MGRN1* is an intracellular C3HC4 RING finger domain protein that exhibits E3 ubiquitin ligase activity and plays critical roles in the control of protein degradation (15). Ubiquitin-mediated proteolysis has played a crucial role in controlling protein level homeostasis and regulating the cell cycle, cell proliferation, apoptosis and DNA damage responses, which are involved in tumorigenesis, tumor development, prognosis and drug resistance (16). However, the role of *MGRN1* in tumorigenesis, tumor progression, and drug responses is not currently well understood. A study by Dugué et al. suggests that hypomethylation of *MGRN1* CpG sites in peripheral blood DNA is associated with the development of sporadic and familiar breast cancer (17).

Based on the results of RRBS, this study investigated the role of hypermethylation of the *MGRN1* upstream region in platinum resistance in HGSOC patients. First, we examined the effects of *MGRN1* methylation status and expression in ovarian tumor samples on the prognosis of HGSOC patients. Furthermore, we also investigated the possible role and mechanism of decreased *MGRN1* expression in ovarian cancer cells in the response to cisplatin *in vitro*. To the best of our knowledge, this is the first study to investigate the role of the methylation status of the *MGRN1* promoter region in platinum resistance in HGSOC patients.

## Materials and Methods

### Tissue Samples

A total of 96 HGSOC tissues were obtained from the Department of Gynecology at the Fourth Hospital, Hebei Medical University, China (November 2011–June 2015). The detailed clinicopathological features of the patients are summarized in **Table 1**. The informed consent of each subject was obtained, and this study was approved by the Medical Ethics Committee of the Fourth Affiliated Hospital of Hebei Medical University. Based on the platinum-free interval (PFI), all the study participants were divided into a platinum-sensitive group (n=55) and a platinum-resistant group (n=41). PFI of less than 6 months is widely used to clinically define platinum-resistant disease, whereas a PFI greater than 6 months is often used to define platinum-sensitive disease (18). The participants were regularly followed-up for 5 years. Overall survival (OS) and progression-free survival (PFS) were used to assess the survival status of the patients.

**Table 1:** Clinical characteristics of 96 HGSOC patients.

Characteristics	Stage	Patients (n)	Recurrence	*p*	Survival	*p*
			HR (95% CI)		HR (95% CI)	
Age	<50 Years	34	Reference	0.71	Reference	0.32
	≥50 Years	62	1.12 (0.74–1.98)		1.12 (0.87–3.11)	
FIGO stage	I-II	21	Reference		Reference	
	III-IV	75	8.73 (1.25–30.15)	0.03	6.66 (1.13–20.12)	0.02
Grade	1	24	Reference		Reference	
	2	42	2.95 (1.75–6.95)	0.05	1.40 (0.37–4.18)	0.30
	3	30	5.40 (1.92–16.01)	0.01	6.40 (0.47–13.90)	0.02
tumor residual size	0	25	Reference		Reference	
	<1cm	48	5.38 (1.95–11.93)	0.01	5.59 (0.37–12.80)	0.02
	>1cm	23	4.09 (1.75–8.99)	<0.01	4.34 (1.35–7.68)	<0.01
*MGRN1*	High expression	35	Reference		Reference	
Expression	Low expression	61	1.48 (0.77–2.83)	0.24	3.12 (1.31–7.11)	0.01
*EGR1*	High expression	22	Reference		Reference	
Expression	Low expression	74	1.39 (0.81-2.97)	0.03	1.66 (0.73-3.21)	0.01

### Genomic DNA Extraction and MALDI-TOF Mass Spectrometry

Of the 96 HGSOC samples, high-quality DNA from 26 HGSOC tissue samples was isolated using the Wizard Genomic DNA Purification Kit (Promega, Madison, Wisconsin), as described by the manufacturers. MALDI-TOF mass spectrometry (Sequenom, San Diego, California, U.S.) was used to detect the methylation level of the *MGRN1* promoter region. This experiment was conducted at CapitalBio Co., Ltd. (Beijing, China). PCR primers were designed using Methprimer (http://www.urogene.org/methprimer). For each reverse primer, an additional T7 promoter tag for *in vivo* transcription was added, whereas a 10 m tag on the forward primer was used to adjust melting temperature differences. Mass spectra were obtained *via* MassARRAY Compact MALDI-TOF (Sequenom). The resultant methylation calls were analysed with EpiTyper software v1.0 (Sequenom) to generate quantitative results for each CpG site or an aggregate of multiple CpG sites.

### RNA Extraction and Quantitative Real-Time Reverse Transcriptase-PCR (RT-qPCR)

Total RNA was isolated from 96 HGSOC tissue samples using the TRIzol-chloroform extraction method (Generay Biotech Co., Ltd., Shanghai, China), as described by the manufacturers. The total cDNA was reverse-transcribed using the Revert Aid First-Strand cDNA Synthesis Kit (Thermo Scientific, USA). The specific primers for the target genes that were used in RT-qPCR were designed using Primer Premier 5.0 and produced by Sangon Biotech Co., Ltd. (Shanghai, China). GAPDH was used as the housekeeping gene. The primer sequences were as follows: *MGRN1* forward, 5’-TACAAAGACGATGCCGACAG-3’; *MGRN1* reverse, 5’-GCCTGGCAGTAGATGGTGAT-3’; *GAPDH* forward, 5’-AATCCC ATCACCATCTTCCA-3’; and *GAPDH* reverse, 5’-TGGACTCCACGA CGTACTCA-3’. The reactions were run with the QuantiNova TMSYBR^®^ Green PCR Kit (Qiagen, Hilden, Germany) in an Mx3005P instrument. The comparative quantification of each target gene was performed based on the cycle threshold (Ct) and normalized to GAPDH using the 2^-ΔCt^ method.

### *MGRN1* Immunohistochemical (IHC) Study of the Clinical Samples

Of the 96 HGSOC samples, 52 paraffin-embedded HGSOC tissue samples collected in the pathology department of the Fourth Hospital of Hebei Medical University were used for immunohistochemical (IHC) staining of MGRN1. MGRN1 immunostaining was performed using a primary antibody, namely rabbit antihuman MGRN1 (RNF156, 1:500 dilution; Proteintech, China). Briefly, 4-μm thick sections were dewaxed in xylene and dehydrated through a graded series of ethanol. After blocking endogenous peroxidase and nonspecific binding, the sections were incubated overnight at 4°C with primary antibody and then with biotinylated secondary antibody and streptavidin-peroxidase complex. After the sections were washed in PBS, they were incubated with DAB reagent and counterstained with haematoxylin. Negative control sections were incubated with PBS instead of primary antibody. The sections were independently examined by two pathologists, who were blinded to the clinicopathological information. The immunoreactivity of MGRN1 was considered to be positive in tumor cells showing cytoplasmic staining without nuclear staining. The immunohistochemical staining was evaluated using a previously reported scoring method (19). If the final score was ≥ 4, the tumor was considered to have high *MGRN1* expression, whereas a score < 4 indicated low *MGRN1* expression.

### Cell Culture

The human serous ovarian cancer SKOV3 cell line was purchased from the iCell Bioscience Inc. (Shanghai, China). The SKOV3 cell line was cultured in Dulbecco’s modified Eagle’s medium (DMEM) (Gibco; Thermo Fisher Scientific, Inc.) The medium was always supplemented with 10% (w/v) fetal bovine serum, 100 U penicillin, and 100 µg/L streptomycin (Gibco; Thermo Fisher Scientific, Inc.). The cells were maintained in a 95% humidified and 5% CO^2^ atmosphere at 37°C. Each vitro experiments were repeat three times biologically.

### Stable Cell Lines

*MGRN1* expression plasmids and lentiviral packaging reagents and shRNA were purchased from Genecopoeia Inc. (MD, USA). The designed three target sequences in the MGRN1 gene were 5′-GGAAACTACTTTGCTTCGCAC-3′ (shRNAa); 5′-GCGTGTTTCCAGTAGTCATC C-3′ (shRNAb) and 5′- GGCATTGAGAACAAGAACAAC-3′ (shRNAc). The most effective construct, recombinant plasmid inserted with MGRN1 gene shRNA expression vector shRNAa was selected for the study. A random sequence of shRNA (shNC) was used as the negative control. Transfection of the SKOV3 cell line was performed according to the manufacturer’s protocol. In brief, SKOV3 cells were seeded in a six-well plate at a density of 4×10^5^ cells/mL in a volume of 2mL/well. When the SKOV3 cells reached 70–80% confluence, they were transfected with shRNA. Forty-eight hours later, culture medium containing 1 μg/ml puromycin (Genecopoeia) was used for selection for one week. The surviving cells were reseeded in fresh culture medium. Then, *MGRN1* gene expression was observed under a fluorescence microscope, and the cells were subjected to RT-qPCR analysis to confirm the downregulation of *MGRN1*.

### Detection of Changes in *MGRN1 via* Western Blotting

Proteins were isolated using RIPA lysis buffer. Total protein was extracted and BCA protein assay kit (Thermo) was used to quantify protein, followed by SDS-PAGE electrophoresis. The protein separated by electrophoresis was electro-transferred onto PVDF membranes, and blocked with 5% skim milk. Then, 1: 1000 rabbit anti-human MGRN1 antibody(RNF156, Proteintech, China) was added into the protein, and the solution was kept at 4°C overnight. The membranes were washed with TBST buffer 3 times (10 min each time). Anti-rabbit IgG was used as the secondary antibody (a dilution of 1: 5000; Proteintech, China), and the solution was cultured for 1h at 37°C. The membranes were washed with TBST for 3 times (10 min each time), and then with TBS for 10min. The antigen-antibody reaction was visualized by detection with Odyssey infrared imaging system (LI-COR Biosciences, Lincoln, NE, USA), and β-actin (ab8226, Abcam, Cambridge, UK) served as internal reference.

### Cell Viability Assays

The cells were inoculated in 96-well microplates in medium containing 10% fetal bovine serum and penicillin/streptomycin. After overnight incubation, the cells were treated with cisplatin (Pfizer), returned to the incubator for 24 h, and then analyzed. Cell Counting Kit-8 (CCK-8) was used to measure cell activity. Ten microliters of CCK-8 was added to each well and incubated for 3 h (37°C; 5% carbon dioxide). Then, the absorbance was measured at 492 nm with a microplate reader. Each vitro experiments were repeat three times biologically.

### Apoptosis Assays

The Annexin V Apoptosis Detection kit I (BD Biosciences, Franklin Lakes, NJ, USA) was used to analyze the apoptosis of the SKOV3 cells. Briefly, the concentration of ovarian cancer cells at logarithmic growth phase was adjusted to 3×105/mL, and the cells were inoculated into 6-well plates at 2 ml/well. The cells were collected after 24 h of cisplatin treatment. After washing with PBS two times, the cells were resuspended in 100 μL of 1 × binding buffer and subsequently incubated with 5 μL of Annexin V staining solution at room temperature for 30 min in the dark. Then, 400 μL of 1 × binding buffer was added, and the cell apoptosis rate of each group was determined by flow cytometry.

### RNA Sequencing

This experiment was conducted at Differential Gene Technology Co., Ltd. (Anhui, China). EdgeR was used to identify the top ten enriched annotation terms among the differentially expressed genes (1.5-fold in either direction, *P*<0.05) between the SKOV3 sh-NC group and the SKOV3 sh-*MGRN1* group.

### Statistical Analysis

The statistical analyses were performed using SPSS 21.0 (Chicago, IL, USA). The Wilcoxon rank sum test was used to compare the methylation level and mRNA expression of *MGRN1* between the two groups. The χ2 test was used to compare the protein expression of *MGRN1* in each group. Spearman correlation analysis was performed to analyze the correlation between *MGRN1* expression and methylation status. Kaplan–Meier analysis and the Cox proportional hazard model were used to analyze the relationship between the methylation level and mRNA expression of *MGRN1* and the prognosis of HGSOC. A t test was used to analyze the cell activity and apoptosis data.

## Results

### *MGRN1*Promoter Methylation Levels in the Platinum-Resistant Group and Platinum-Sensitive Group

**Figure 1:**
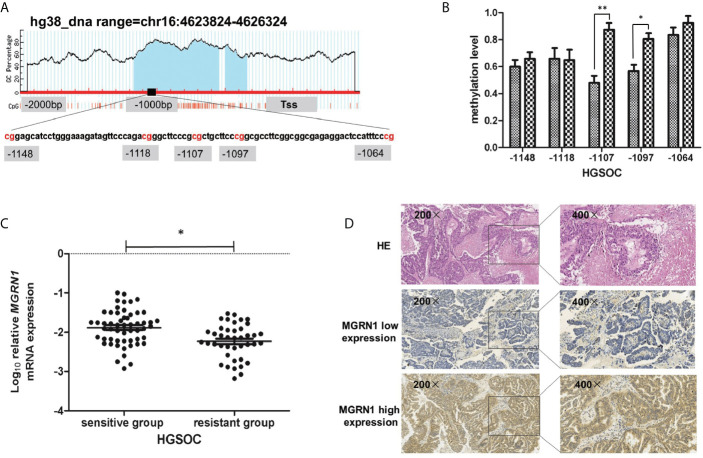
The methylation level of *MGRN1* promoter was associated with platinum-resistant in HGSOC patients. **(A)** Schematic diagram of the abnormal methylation region (-1148 to -1064 upstream) in the promoter region of the *MGRN1* gene. **(B)** The methylation of -1107 and -1097 CpG site were significantly hypermethylated in platinum-resistant HGSOC tissues compared with platinum-sensitive HGSOC tissues by MALDI-TOF Sequenom MassARRAY. **(C)** The mRNA expression of *MGRN1* was lower in platinum-resistant HGSOC patients than platinum-sensitive HGSOC patients. **(D)** MGRN1 protein expression in HGSOC tissues. *P < 0.05, **P < 0.01.

In our previous study, we used the RRBS assay to compare the differences in the genome-wide methylation patterns between 8 platinum-resistant epithelial ovarian cancer patients and 8 platinum-sensitive epithelial ovarian cancer patients. The results showed that a region from -1148 to -1064 within the promoter of *MGRN1* was significantly hypermethylated in the platinum-resistant group compared to the platinum-sensitive group (**Figure 1A**). To further confirm the results of the RRBS assay, MALDI-TOF mass spectrometry was used to examine the methylation levels of this region in 12 platinum-resistant HGSOC patients and 14 platinum-sensitive HGSOC patients. In the present study, we tested the methylation levels of five CpG sites (-1148, -1118, -1107, -1097 and -1064 from the transcription start site) within this region. The analysis revealed that methylation levels of two CpG sites (-1107 and -1097) were significantly higher in the tumor tissues of platinum-resistant HGSOC patients than in those of platinum-sensitive HGSOC patients (*P*=0.01, 0.04, **Figure 1B**).

### MGRN1 Expression in the Platinum-Resistant Group and Platinum-*S*ensitive Group

RT-qPCR was used to determine the mRNA levels of *MGRN1* in the tumor tissues from 41 platinum-resistant HGSOC patients and 55 platinum-sensitive HGSOC patients. The results showed that the mRNA level of *MGRN1* in platinum-resistant HGSOC patients was 1.20-fold lower than that in platinum-sensitive HGSOC patients (*P*=0.01, **Figure 1C**). Furthermore, IHC analysis was conducted to examine the protein expression of MGRN1 in 25 platinum-resistant HGSOC patients and 27 platinum-sensitive HGSOC patients. The analysis results showed that the frequency of positive MGRN1 expression in platinum-resistant HGSOC patients was significantly lower than that in platinum-sensitive HGSOC patients (*P*=0.02, **Table 2**). IHC staining showed that the MGRN1 protein was mainly expressed in the cytoplasm of HGSOC tissues (**Figure 1D**).

**Table 2:** Comparison of MGRN1 protein expression between platinum-resistant HGSOC tissues and platinum-sensitive HGSOC tissues.

MGRN1 expression	Resistant group n (%)	Sensitive group n (%)	P
High	15(60.0)	23(85.2)	**0.02**
Low	10(40.0)	4(14.8)	

### Association Between *MGRN1* mRNA Expression and Its Methylation Levels in HGSOC

Spearman’s correlation analysis showed that *MGRN1* mRNA expression was significantly negatively correlated with the methylation level of the *MGRN1* promoter region (average of -1107/-1097 CpGs: r=-.511, *P*=0.01). The results indicated that the hypermethylation of the *MGRN1* promoter may be responsible for the downregulation of *MGRN1* mRNA expression in HGSOC tissues.

### Silencing of *MGRN1* Expression in Serous Ovarian Cancer Cells by shRNA

**Figure 2:**
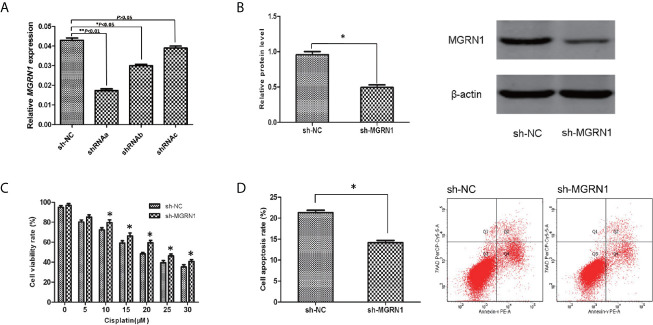
The alteration of the sensitivity to cisplatin after a knockdown of *MGRN1* expression in SKOV3 cells. **(A)** RT-qPCR assay showed the reduced expression of *MGRN1* in shRNAa-*MGRN1*, shRNAb-*MGRN1* and shRNAc-*MGRN1* cells compared to shNC cells. **(B)** Western blot assay showed the reduced expression of *MGRN1* in shRNAa-*MGRN1* cells compared to shNC cells. **(C)** CCK-8 assays showed a significant increase in the proliferation rates in the shRNA-*MGRN1* cells compared with the shNC cells after cisplatin treatment at several concentrations for 24h. **(D)** Flow cytometry showed that the apoptosis rate in the shRNA-*MGRN1* cells was significantly lower than that in the shNC cells after exposure to cisplatin at the 10μM concentration for 24h. *P < 0.05, **P < 0.01. All the experiments were performed in triplicate.

To investigate the role of *MGRN1* expression in the sensitivity of serous ovarian cancer cells to cisplatin, SKOV3 cells were transfected with shRNAa-*MGRN1*, shRNAb-*MGRN1*, shRNAc-*MGRN1* plasmid or shNC plasmid, respectively. After transfection for 48 hours, the expression of *MGRN1* was confirmed by RT-qPCR. As shown in **Figure 2A**, shRNAa-*MGRN1* could effectively decrease *MGRN1* expression in SKOV3 cells, as compared with shNC groups. We also confirmed the expression of *MGRN1* in shRNAa-*MGRN1* group by Western blot. Therefore, we established *MGRN1* stable knockdown cell lines using shRNAa-*MGRN1* (*P*<0.05, **Figures 2A, B**).

### Effect of *MGRN1* Knockdown on the Cellular Response to Cisplatin

CCK-8 assays were used to compare the cell proliferation of the shRNA-*MGRN1* group and shNC group. The proliferation rate was significantly higher in the shRNA-*MGRN1* group than in the shNC group after treatment with cisplatin at several concentrations for 24 h (*P*<0.05, **Figure 2C**). In addition, flow cytometry analysis demonstrated that the percentage of apoptotic cells in the shRNA-*MGRN1* group was significantly lower than that in the shNC group after exposure to 10 μM cisplatin (*P* =0.03, **Figure 2D**).

### RNA-Seq Analysis Reveals That *EGR1* Expression Is Differentially Regulated by *MGRN1* in Ovarian Cancer Cells

**Figure 3:**
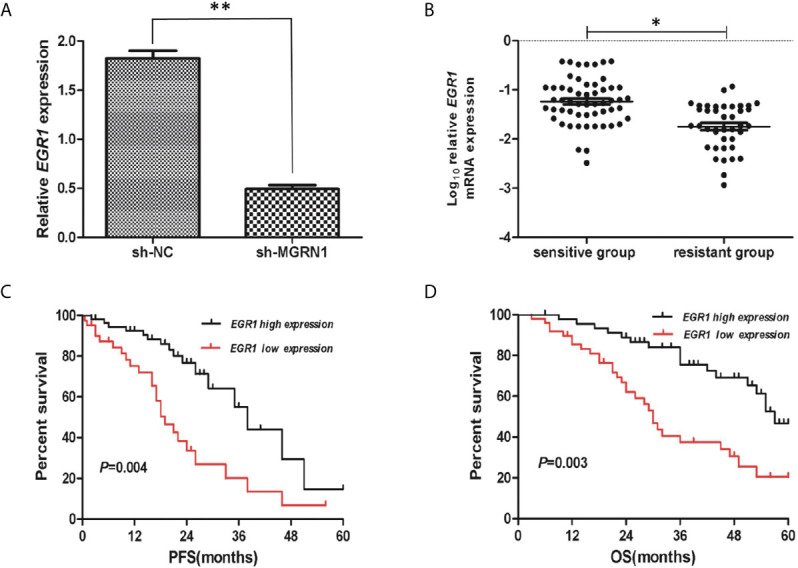
The low mRNA expression of *EGR1* was associated with platinum-resistant in HGSOC patients. **(A)** RT-qPCR showed the reduced expression of *EGR1* in sh-*MGRN1* cells compared to shNC cells. **(B)** The mRNA expression of *EGR1* in platinum-resistant HGSOC patients and platinum-sensitive HGSOC patients. **(C, D)** Kaplan-Meier analysis of PFS and OS according to the *EGR1* mRNA expression in HGSOC patients. *P < 0.05, **P < 0.01.

To gain a better understanding of the differential regulation of transcription between the shNC transfection group and shRNA-*MGRN1* transfection group, we performed an RNA-seq analysis of the total RNA harvested from the shNC group and shRNA-*MGRN1* group. The top ten annotated, protein-coding genes that were differentially regulated in the shNC group compared to the shRNA-*MGRN1* group are shown in **Table 3** (*P*<0.05). Of these genes, *EGR1* was the most differentially expressed, with 4.26-fold lower expression in the shRNA-*MGRN1* group than in the shNC group (*P*=8.65E^-07^). *EGR1* is a key gene involved in regulating cell proliferation and apoptosis in a variety of cancer tissues, and knockdown of *EGR1* has been shown to promote resistance to cisplatin. Thus, we further validated the mRNA levels of *EGR1* in cells and tissues by quantitative reverse-transcription PCR (RT-qPCR). The results showed that *EGR1* mRNA expression was reduced by 72% in the SKOV3 shRNA-*MGRN1* group compared with the SKOV3 shNC group (*P*<0.01, **Figure 3A**). The mRNA level of *EGR1* in 41 platinum-resistant HGSOC patients was 1.15-fold lower than that in 55 platinum-sensitive HGSOC patients (*P*=0.02, **Figure 3B**). Spearman’s correlation analysis showed that *MGRN1* mRNA expression was significantly positively correlated with *EGR1* mRNA expression (r=-.379, *P*=0.01). Next, we used publicly available ovarian cancer data from The Cancer Genome Atlas (TCGA) series to validate our results. *MGRN1* mRNA expression was also strongly associated with *EGR1* mRNA expression in the ovarian cancer population (*P*<0.01).

**Table 3:** Genes differentially expressed between shRNA-*MGRN1* group and shNC group.

Gene symbol	Description	Fold change	*p*
FOSB	FosB Proto-Oncogene, AP-1 Transcription Factor Subunit	-6.51	5.00E-08
EGR1	Early Growth Response 1	-4.26	8.65E-07
NGFR	Nerve Growth Factor Receptor	-5.53	0.000002
ADM	Adrenomedullin	-3.25	0.00009
KRT5	Keratin 5	-4.44	0.00010
HK2	Hexokinase 2	-3.23	0.00011
CES1P2	Carboxylesterase 1 Pseudogene 2	8.9	0.00015
DEPP1	DEPP1 Autophagy Regulator	-4.26	0.00024
STC1	Stanniocalcin 1	-3.6	0.00026
EDIL3	EGF Like Repeats And Discoidin Domains 3	-4.71	0.00033

### Hypermethylation and Low Expression of *MGRN1* Correlate With Poor Prognosis of HGSOC Patients

**Figure 4:**
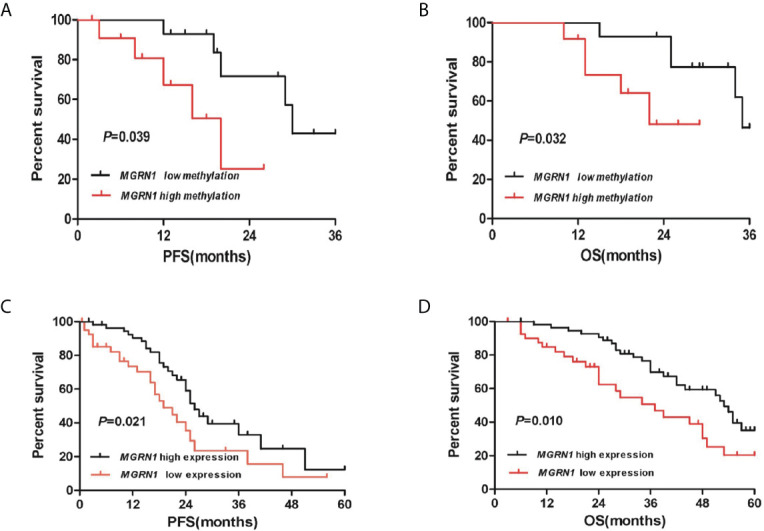
The high methylation of *MGRN1* and its low mRNA expression are associated with poor survival in HGSOC patients. **(A, B)** Kaplan-Meier analysis of PFS and OS according to the *MGRN1* methylation level in 26 HGSOC patients. **(C, D)** Kaplan-Meier analysis of PFS and OS according to the *MGRN1* mRNA expression in 96 HGSOC patients.

A total of 26 HGSOC patients were divided into hypermethylation and hypomethylation groups based on the median value of *MGRN1* methylation. Kaplan-Meier survival analysis revealed that the *MGRN1* hypermethylation group exhibited lower PFS and OS of HGSOC patients compared to the *MGRN1* hypomethylation group (*P*=0.04, **Figure 4A**; *P*=0.03, **Figure 4B**). Next, we investigated the correlation between *MGRN1* mRNA expression and the clinical outcomes of HGSOC patients. A total of 96 HGSOC patients were divided into low- and high-expression groups based on the median value of *MGRN1* mRNA expression. Kaplan-Meier analysis showed that compared with high *MGRN1* expression, low *MGRN1* expression was associated with significantly lower PFS and OS of HGSOC patients (*P*=0.02, **Figure 4C**; *P*=0.01, **Figure 4D**). After adjusting for other prognostic factors (age, stage, grade and tumor residual size), low *MGRN1* expression was also significantly associated with shorter OS (**Table 1**, *P*=0.01), demonstrating that *MGRN1* expression was an independent predictor of poorer clinical outcomes in HGSOC patients. Furthermore, Kaplan-Meier analysis demonstrated that the low *EGR1* expression group was associated with worse prognosis compared to the high *EGR1* expression group (*P*<0.01, **Figures 3C, D**). Multivariable analysis showed that *EGR1* expression was an independent predictor of worse clinical outcomes in HGSOC patients (**Table 1**, *P*=0.03, 0.01).

## Discussion

In this study, we confirmed that *MGRN1* gene promoter hypermethylation is associated with platinum resistance in patients with HGSOC based on the following findings: 1) the upstream region of *MGRN1* was significantly hypermethylated in the cancer tissues of platinum-resistant patients with HGSOC, 2) the lower expression of *MGRN1* due to hypermethylation of the upstream region was associated with clinical outcomes of patients with HGSOC, 3) knockdown of *MGRN1* expression could desensitize SKOV3 ovarian cancer cells to cisplatin, 4) knockdown of *MGRN1* expression in SKOV3 cells could significantly reduce *EGR1* mRNA expression, which significantly correlated with the treatment outcomes in cisplatin-treated cancer patients, and 5) *EGR1* expression in the cancer tissues of platinum-resistant patients was significantly lower than that of platinum-sensitive patients and was related to the clinical prognosis of patients with HGSOC.

Based on the results of previous RRBS analysis, we found that the methylation level of the *MGRN1* upstream region (-1148 to -1064) was significantly higher in the platinum-resistant HGSOC patients. Mass spectrometry further analysis showed that hypermethylation of the *MGRN1* promoter region was associated with platinum resistance in HGSOC patients. We also discovered that the expression levels of *MGRN1* mRNA and protein in platinum-resistant HGSOC patients were significantly lower than those in platinum-sensitive HGSOC patients. Correlation analysis indicated that the methylation level of the *MGRN1* promoter region was associated with *MGRN1* mRNA expression. Furthermore, knockdown of *MGRN1* expression could increase proliferation and decrease apoptosis in SKOV3 cells challenged with cisplatin. These findings suggested that lower *MGRN1* expression due to hypermethylation of its promoter region might induce platinum resistance in HGSOC.

*MGRN1*, an E3 ubiquitin ligase of the Really Interesting New Gene (RING) finger family, is involved in many biological and cellular mechanisms (20). However, there is no study of the role of *MGRN1* in chemotherapy resistance in cancer patients to date. In the current study, microarray analysis of total RNA showed that knockdown of *MGRN1* expression in SKOV3 cells resulted in significant downregulation of multiple genes, including early growth response protein 1 (*EGR1*). *EGR1* is a transcription factor that can be induced by a variety of stimuli or stressors, including growth factors, hormones, ionizing radiation, and chemotherapy drugs (21–23), and plays essential roles in cell proliferation and apoptosis (24–26). Knockdown of *EGR1* expression can decrease cisplatin-induced apoptosis in a variety of cancer cells (27–29), while overexpression of this gene sensitizes ovarian cancer cells to cisplatin-induced apoptosis (28). He et al. found that *EGR1* expression levels were significantly higher in ovarian cancer tissues with low *ERCC1* expression than in ovarian cancer tissues with high *ERCC1* expression, suggesting that *EGR1* expression is positively correlated with potential cisplatin-sensitive ovarian cancer, since *ERCC1* is widely accepted as a biomarker of platinum resistance (28). In our study, it was also observed that the expression of *EGR1* in HGSOC patients with platinum resistance was significantly downregulated and was positively correlated with the expression of *MGRN1*. Although the mechanism by which *MGRN1* regulates *EGR1* is still unclear, a strong association between *MGRN1* mRNA expression and *EGR1* mRNA expression was noted in the TCGA ovarian cancer dataset (**Additional File 1** and **Figure S1**). Therefore, we speculate that *MGRN1* may affect the platinum resistance of ovarian cancer by regulating the expression of *EGR1*. Of course, the molecular mechanism by which *MGRN1* regulates *EGR1* requires further study.

More importantly, Kaplan-Meier analyses showed that hypermethylation of the *MGRN1* promoter region was associated with worse survival of HGSOC patients in this study. Further, multivariable analysis also indicated that HGSOC patients with lower *MGRN1* mRNA expression have a worse prognosis than those with higher *MGRN1* mRNA expression, which suggested the prognostic value of *MGRN1* methylation for HGSOC patients due to the negative correlation between *MGRN1* methylation and its expression. Here, we also confirmed that patients with low *EGR1* mRNA expression had significantly shorter PFS and OS than those with high *EGR1* mRNA expression. Of note, data derived from TCGA showed that the expression of *EGR1* significantly correlated with the treatment outcomes in cisplatin-treated cancer patients (30). Therefore, *EGR1* expression may be a useful marker for predicting the clinical prognosis of HGSOC patients.

In summary, our study demonstrated that the hypermethylation of *MGRN1* is an independent marker of poor prognosis in HGSOC patients and may also be predictive of platinum resistance in HGSOC patients. Considering that DNA methylation may be used as a molecular marker for ovarian cancer chemotherapy, we believe that our findings warrant confirmation in a larger patient cohort and could facilitate patient selection for chemotherapy.

## Data Availability Statement

The original contributions presented in the study are included in the article/**Supplementary Material**. Further inquiries can be directed to the corresponding authors.

## Ethics Statement

The studies involving human participants were reviewed and approved by the institute Medical Ethics Committee of Hebei Medical University, Fourth Hospital.

## Author Contributions

Administrative support: X-FL, SK, and YL. Provision of study materials or patients: X-FL and H-YS. Collection and assembly of data: X-FL and TH. Data analysis and interpretation: X-FL, H-BZ, and Y-JT. All authors contributed to the article and approved the submitted version.

## Conflict of Interest

The authors declare that the research was conducted in the absence of any commercial or financial relationships that could be construed as a potential conflict of interest.

## Supplementary Material

Supplementary Figure 1 The association between *MGRN1* mRNA expression and *EGR1* mRNA expression in the TCGA ovarian cancer dataset.
