# Supplementary figures and images for: Promoter Methylation of the MGRN1 Gene Predicts Prognosis and Response to Chemotherapy of High-Grade Serous Ovarian Cancer Patients
Source: Front Oncol. 2021 Jun 29;11:659254. doi: 10.3389/fonc.2021.659254 (PMC8277380; doi:10.3389/fonc.2021.659254)

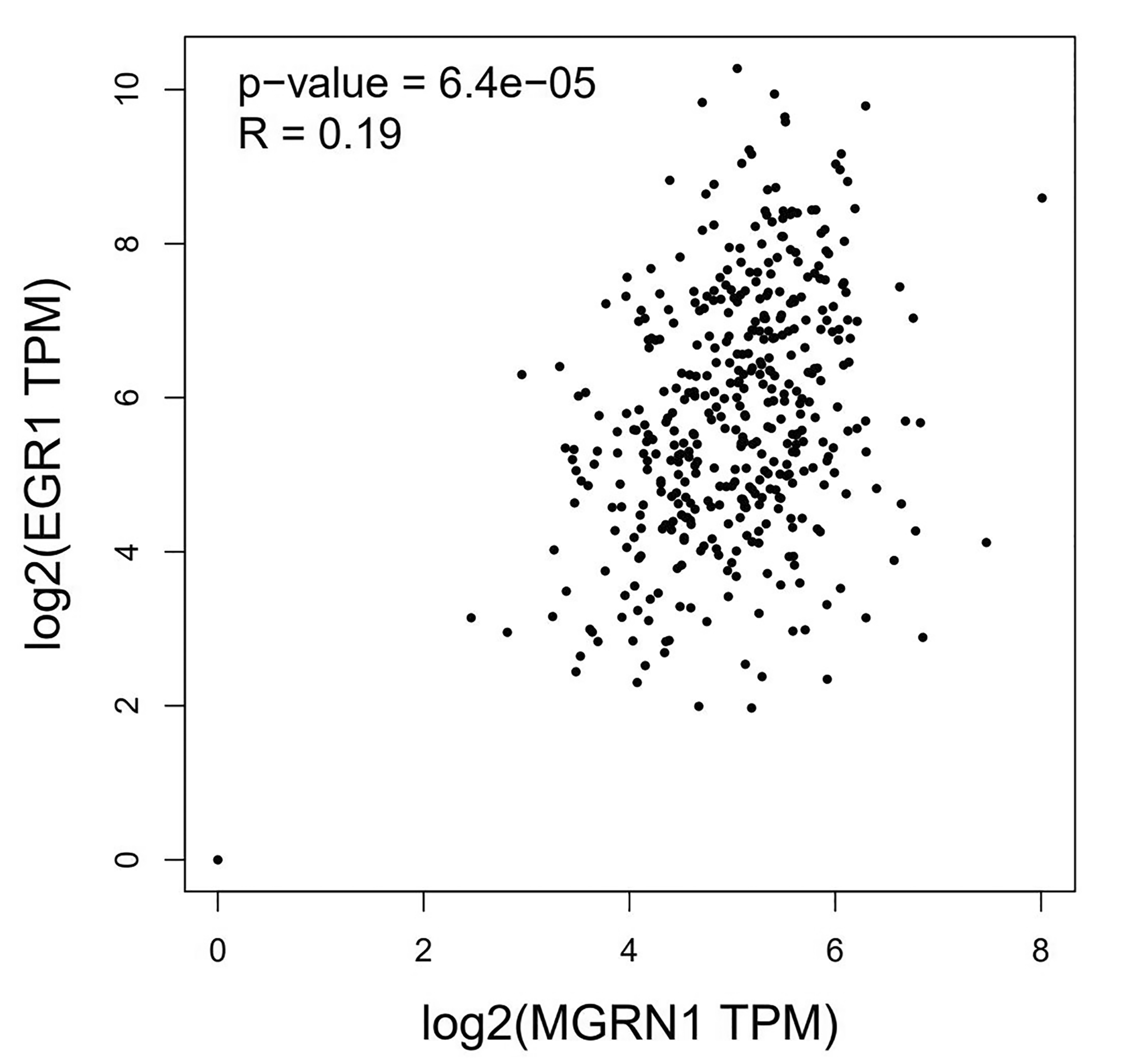

Supplement: Supplementary Figure 1 — The association between MGRN1 mRNA expression and EGR1 mRNA expression in the TCGA ovarian cancer dataset. [file Image_1.tif]
